# HOW AND WHY TO APPLY FOR GERONTOLOGY ACCREDITATION

**DOI:** 10.1093/geroni/igz038.1470

**Published:** 2019-11-08

**Authors:** Janet S Hahn, Donna E Schafer

**Affiliations:** 1 Western Michigan University, Kalamazoo, Michigan, United States; 2 National Association for Professional Gerontologists, Healdsburg, California, United States

## Abstract

This presentation covers the steps to achieve accreditation of a gerontology program as well as the costs and benefits of accreditation. The overall timeline for a typical accreditation process is presented as well as the organization of required standards. Advance planning and a close review of Accreditation for Gerontology Education Council standards will allow time for a program to comply and document compliance with standards. Communication and cooperation within an institution are needed for a program to successfully seek accreditation. Key individuals and important conversations will be identified to assist those who are considering accreditation for their gerontology program.

